# Lateral External-fixation Adjacent to Radial Nerve

**DOI:** 10.7759/cureus.7435

**Published:** 2020-03-27

**Authors:** Benjamin F Plucknette, David J Tennent, Joseph R Hsu, Taylor Bates, Travis C Burns

**Affiliations:** 1 Orthopaedic Surgery, San Antonio Military Medical Center, San Antonio, USA; 2 Orthopaedic Surgery, Carolinas Medical Center, Charlotte, USA

**Keywords:** upper extremity external fixation, radial nerve, external fixation, combat trauma

## Abstract

Introduction

The aim of our study was to describe the injury pattern and outcomes of active-duty subjects that underwent humeral external fixation and to determine if the placement of external fixator pins outside of the radial nerve safe zones is correlated with injury to the radial nerve.

Materials and methods

We examined all US Service members treated with humeral external fixation at our facility from June 2005 through June 2015. The mechanism of injury, injury pattern, location of external fixation application, pre- and postoperative radial nerve function, presence or absence of radial nerve transection from injury or external fixation, anatomic location of pins in relation to the radial nerve safe zone, and final radial nerve outcomes were recorded. We defined the proximal safe zone as 5 cm distal to the acromion to 14.8 cm proximal to the lateral epicondyle, and we defined the distal safe zone as the proximal 70% of the transepicondylar width of the humerus when projected proximally from the lateral epicondyle.

Results

For our study, 123 patients were identified over our date range, and 16 subjects were included with documentation regarding nerve function/injury characteristics, appropriate radiographs, and active duty status. Around 80% of injuries resulted from a blast mechanism, and 80% of injury patterns included either an intraarticular or open fracture. The radial nerve safe zone was violated in 15 of the 16 subjects (94%). The one subject with a safe construct did not sustain a nerve injury. Complete preoperative documentation on nerve function was only available for half of the subjects. Two of five subjects known to have intact function prior to external fixation had a postoperative neurologic deficit (40%). Of eight subjects with unknown radial nerve function prior to external fixation, seven subjects had full nerve function at the final follow up, and one subject had partial sensory function only. Of the three subjects with impaired preoperative radial nerve function, two made a full recovery, and the third recovered sensory function only. Around 50% of all subjects required medical retirement.

Conclusion

External fixation of upper extremity injuries in combat is rarely absolutely indicated, often results in the placement of pins outside of the radial nerve safe zone, and is associated with up to a 40% incidence of radial nerve injury.

## Introduction

External fixation is used for stabilization of combat-related complex humerus fractures in forward surgical settings [1]. Some authors consider external fixation as a definitive treatment option for these high-energy fractures [2]. Iatrogenic injury to the radial nerve continues to be a primary concern when placing the external fixation pins. One study found that 10% of external fixator pins penetrated or contacted the radial nerve [3]. The radial nerve courses posterior to the humerus from 20.7 +/- 1.2 cm proximal to the medial epicondyle to 14.2 +/- 0.6 cm proximal to the lateral epicondyle before piercing the lateral intermuscular septum 10.2 +/- 0.4 cm proximal to the lateral epicondyle [4]. Various safe zones for pin entry about the distal humerus have been described in relation to the radial nerve. Kamineni et al. described a safe zone about the radial nerve based on the patient’s transepicondylar axis [5]. The distance across the distal humerus is measured from the tip of the medial epicondyle to the tip of the lateral epicondyle. Once the transepicondylar width is known, this same distance is projected superiorly from the lateral epicondyle. The superior 70% of this projection was considered a safe zone. 

The literature is devoid of absolute indications for upper extremity external fixation, but commonly cited indications include vascular injury, unstable elbow dislocations, and unstable grade III open fractures [6]. The aim of our study was to determine the rate of temporary external fixator pin placement outside of the described safe zones, describe the functional status of the radial nerve both pre- and postoperatively, and to report the military-specific outcomes of patients after external fixation about the humerus.

## Materials and methods

The Surgical Scheduling System at our institution, the Department of Defense’s only Level 1 trauma center, was queried for all upper extremity external fixation applications or removals from June 2005 through June 2015. Of the subjects identified by this search, only active duty subjects were included. We then viewed individual radiographs to evaluate each subject for appropriate radiographs of the involved upper extremity. 

**Figure 1:**
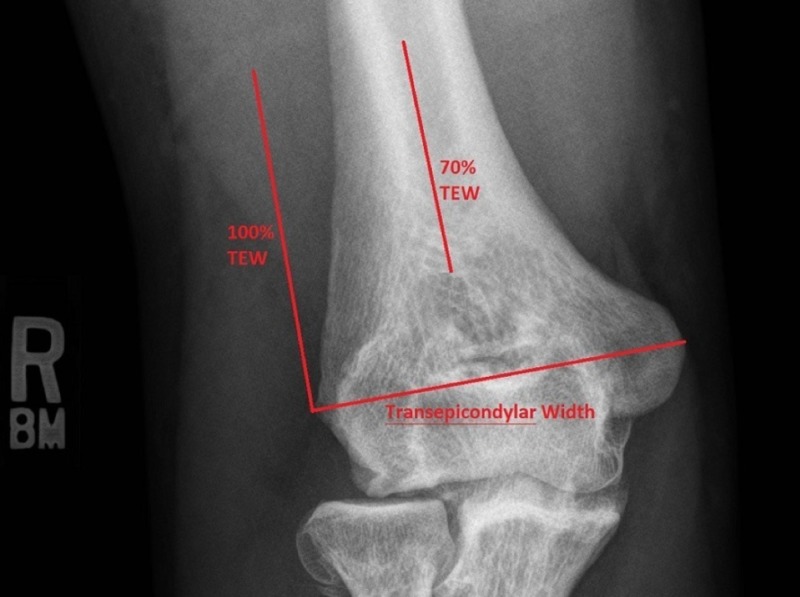
Measurement and application of transepicondylar width (TEW)

For each radiograph, fractures were described as either intraarticular or extra-articular, and associated fractures of the ulna or radius were noted. For the external fixator construct, the number of pins and their locations were documented. Pin locations were described in millimeters superior to both the medial and lateral epicondyles individually. The transepicondylar width (TEW) was also documented by measuring the transverse distance between the peak of the medial and lateral epicondyles, and each subject’s specific distal humeral safe zone was calculated based on this measurement. The distal humeral safe zone was defined as the superior 70% of a line equal in length to the TEW that was extended superiorly from the lateral epicondyle, as described by Kamineni (Figure 1) [5]. 

**Figure 2:**
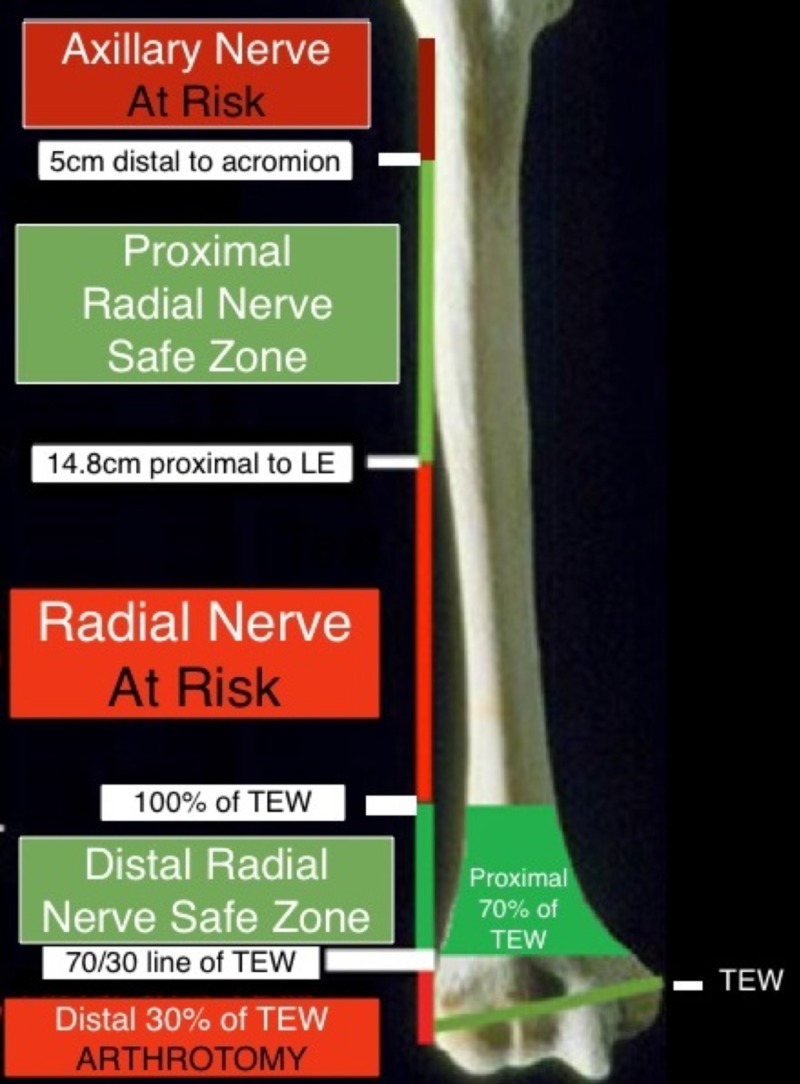
Radial nerve safe zones about the lateral humerus

An additional safe zone exists superiorly about the humerus based on cadaveric studies by Gerwin, wherein the radial nerve was determined to cross the lateral humerus at an average of 14.2 +/- 0.6cm superior to the lateral epicondyle [4]. This more proximal safe zone extends along the lateral humerus from 5 cm distal to the acromion (the region where the axillary nerve lies) to 14.8 cm proximal to the lateral epicondyle (the upper limit of Gerwin’s confidence interval for where the radial nerve crosses the lateral humerus). The two safe zones are demonstrated together in Figure 2. Any violation of the radial nerve safe zone was documented, and placement of pins outside of these zones was considered to place the radial nerve at risk for injury.

For subjects with appropriate radiographs and all required measurements, our electronic medical record was queried for information regarding the injury, surgery, and outcome. Associated upper extremity injuries to include neurovascular injuries and elbow dislocations were documented. Specifically, clinical notes were reviewed for pre- and postoperative documentation of radial nerve sensory and motor function. Additionally, operative reports were reviewed for the documented complication of an injury to the radial nerve. Subjects with radial nerve injury or transections documented as part of the injury pattern were excluded. External fixator data recorded included location of application and date of application. Outcomes were determined by documenting immediate postoperative radial nerve function, date of external fixator removal, date of final follow up, final radial nerve sensory outcome, final radial nerve motor outcome, and military disposition (continued active duty or medically retired). Subjects were excluded if they did not have at least three months of postoperative follow up, if they lacked sufficient documentation, or if they were noted to have a radial nerve transection not related to external fixator placement.

## Results

Within our surgical scheduling system, 123 subjects were identified to have undergone upper extremity external fixator placement, revision, or removal. Of these subjects, 16 were found to be active-duty service members with available radiographs and clinical documentation that met study inclusion criteria.

All injuries were sustained in a deployed setting. Mechanisms included 13 of 16 injuries secondary to blast, and three of 16 of injuries secondary to gunshot wounds (GSW). A single subject sustained a brachial artery injury associated with a GSW. Elbow dislocations were noted in five subjects, which were all associated with blast injuries except for one associated with a GSW. There were four intraarticular humerus fractures, one intra-articular radius fracture, and four intraarticular ulna fractures. Around 75% of injury patterns included an open fracture. Injury information for all of the 16 cases is contained in Table 1.

**Table 1:** Injury characteristics GSW – gunshot wound

Preoperative neurological Status	Total	Blast	GSW	Intraarticular humerus	Intraarticular radius	Intra-articular ulna	Open humerus	Open radius	Open ulna
Unknown	8	6	2	1	0	4	4	0	4
Intact	5	4	1	2	0	0	4	1	1
Impaired	3	3	0	1	1	0	2	0	1

**Figure 3:**
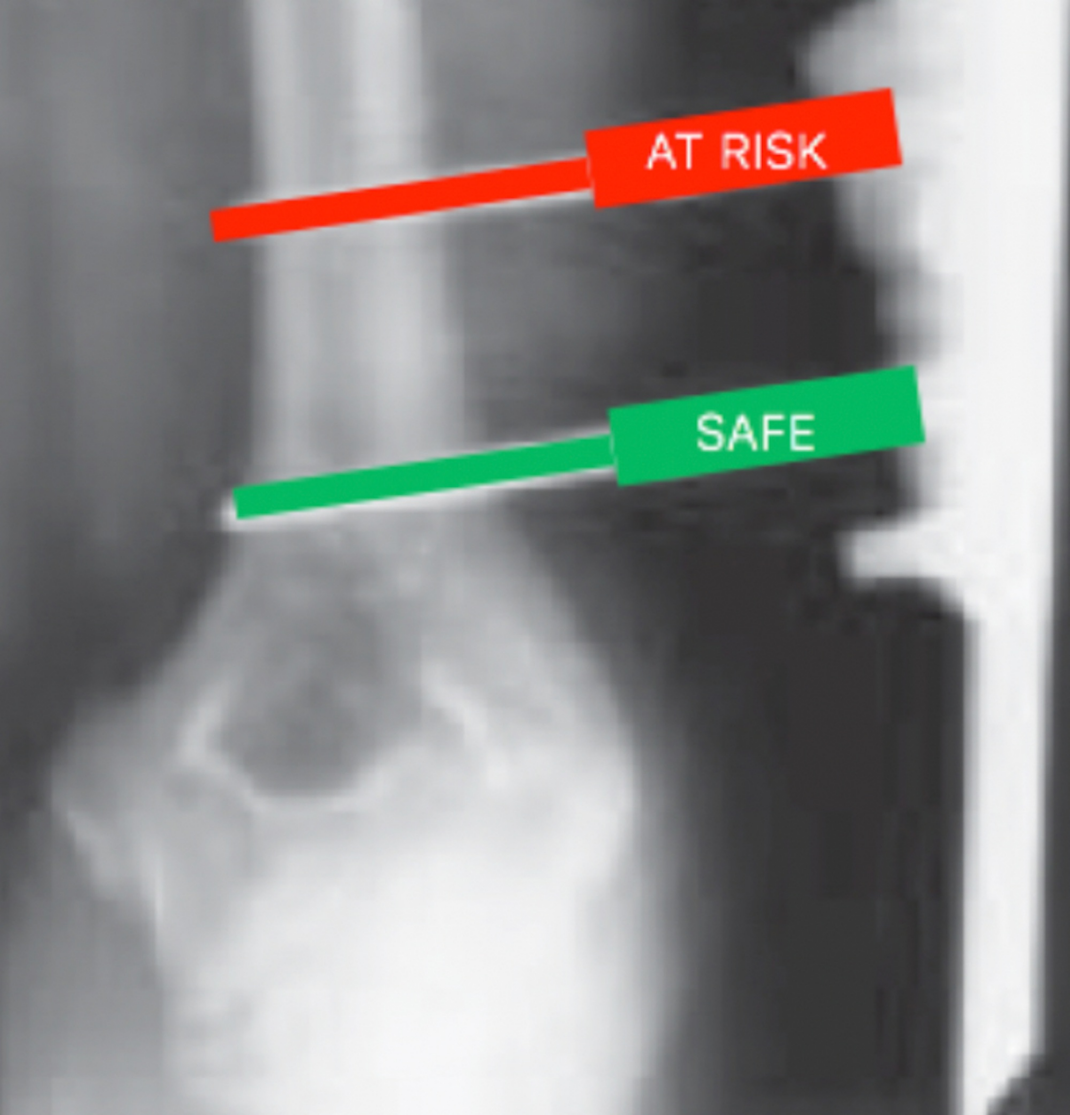
Placement of pins within safe and at-risk zones

Radial nerve function was intact preoperatively in five of 16 subjects (31%), impaired preoperatively in three of 16 subjects (19%), and not documented preoperatively in eight of 16 subjects (50%). Information regarding pin placement and neurologic status is included in Table 2. External fixators were applied in a forward deployed setting in 11 of 16 (69%) subjects, while five of 16 (31%) external fixators were applied in larger echelon military treatment facilities in Europe and America. External fixator pins were placed outside of the radial nerve safe zone (in an area that placed the radial nerve at risk) in 15 of 16 (94%) constructs. Of this 94%, 38% of the subjects had both pins placed outside of the safe zones. When only one of the two pins was placed in the safe zone, it was always the more proximal pin that endangered the nerve (Figure 3). 

**Table 2:** Pin placement in relation to individual TEW measurements and neuro outcomes TEW – transepicondylar width; FOB – Forward operating base (deployed setting); BAMC – Brooke Army Medical Center (San Antonio, TX); LRMC – Landstuhl Regional Medical Center (Germany); GSW – gunshot wound; RTD – return to duty

Subject	Location of application	MOI	Intra-articular fracture	Open Fracture(s)	Preoperative radial nerve function	Proximal pin (mm)	Distal pin (mm)	TEW (mm)	Postoperative radial nerve function	Sensory outcome	Motor outcome	Disposition	Safe zone (mm)	No. of Safe pins
1	FOB	Blast	None	Humerus	Unknown	74	55	68	Impaired	Partial	None	Retired	20.4-68	1
2	FOB	Blast	None	Humerus	Impaired	73	42	68	Impaired	Full	Partial	Unknown	20.4-68	1
3	FOB	Blast	None	Humerus	Unknown	96	54	65	Intact	Full	Full	Retired	19.5-65	1
4	BAMC	Blast	None	None	Intact	70	38	60	Intact	Full	Full	Retired	18-60	1
5	BAMC	Blast	None	None	Unknown	64	14	67	Intact	Full	Full	Retired	20.1-67	1
6	BAMC	Blast	Radius/Ulna	None	Impaired	75	53	62	Intact	Full	Full	RTD	18.6-62	1
7	LRMC	Blast	Humerus	Humerus/Radius	Intact	135	102	73	Intact	Full	Full	Unknown	21.9-73	0
8	FOB	GSW	None	Humerus/Ulna	Unknown	82	47	67	Intact	Full	Full	Retired	20.1-67	1
9	FOB	GSW	None	Humerus	Intact	110	79	77	Impaired	None	None	RTD	23.1-77	0
10	FOB	Blast	Humerus/Ulna	Humerus/Ulna	Intact	123	104	66	Impaired	Full	Full	Retired	19.8-66	0
11	FOB	Blast	None	Humerus	Intact	102	61	72	Intact	Full	Full	RTD	21.6-72	1
12	FOB	Blast	Humerus	Humerus/Ulna	Impaired	172	124	72	Impaired	Full	Full	RTD	21.6-72	1
13	BAMC	Blast	Ulna	Ulna	Unknown	55	42	59	Intact	Full	Full	Retired	17.7-59	2
14	FOB	Blast	Humerus/Ulna	Humerus/Ulna	Unknown	124	97	60	Intact	Full	Full	Retired	18-60	0
15	FOB	Blast	None	None	Unknown	143	98	60	Intact	Full	Full	Unknown	18-60	0
16	FOB	Blast	None	Ulna	Unknown	122	82	68	Intact	Full	Full	RTD	20-68	0

Intact radial nerve function was documented to be present in the immediate postoperative period in 11 of 16 (69%) subjects, and all of these subjects had at least one pin placed outside of the safe zones. Of the five subjects with documented radial nerve function prior to surgery, two subjects had deficits in the immediate postoperative period. These two subjects had both distal pins placed outside of the safe zones. This represents a 40% rate of radial nerve injury within this subgroup. At the time of the final follow up, 13 of 16 subjects (81%) had full radial nerve function. The three subjects that lacked full radial nerve function represented one subject each from the unknown, intact, and impaired preoperative nerve function groups. Of these three subjects, two had partial or absent sensation, and all three subjects had partial or absent motor function. Nerve function throughout the treatment period is recorded in Table 3. 

**Table 3:** Nerve function throughout the duration of treatment

Preoperative neurological status	Total	Postoperative neuro Intact	Postoperative neuro impaired	Pin(s) placed outside safe zone	Motor intact at follow-up	Sensory intact at follow-up
Unknown	8	7 (88%)	1 (12%)	7 (88%)	7 (88%)	7 (88%)
Intact	5	3 (60%)	2 (40%)	5 (100%)	4 (80%)	4 (80%)
Impaired	3	1 (33%)	2 (67%)	3 (100%)	2 (66%)	3 (100%)

Final outcomes as determined by military disposition demonstrated eight of the subjects (50%) were medically retired, while five subjects (31%) returned to duty, and three subjects’ final disposition was undetermined or unavailable.

## Discussion

In this study, 123 subjects were identified to have undergone upper extremity external fixator placement, revision, or removal. However, only 16 were found to be active duty service members met study inclusion criteria. The majority (94%) of upper extremity external fixation constructs had a pin placed outside of the radial nerve safe zones. The one subject with a construct observed to be completely within the safe zone had intact nerve function throughout their care. The distal humeral safe zone for pin placement described by Kamineni et al. requires pins to be placed more distal than nearly all observed constructs [5]. In this study, subjects’ injury patterns often precluded safe pin placement because the fracture or zone of injury is often, itself, within the safe zone. Additionally, placing pins distal enough to be within the safe zone requires close proximity to the elbow joint capsule.

Despite the large number of patients initially identified with humeral external-fixation (123), sufficient imaging and follow up for inclusion was rare. Only 16 subjects could be included in the study, and only five of these 16 had documented preoperative radial nerve function-with the remaining 11 subjects’ preoperative nerve function either impaired (three) or unknown (eight). Thus, the study is most useful when the results are interpreted separately for the entire cohort and the subgroup with known preoperative nerve function. The subgroup includes the five of 16 subjects with documented preoperative radial nerve function, which can demonstrate the humeral external fixator’s causation in the rate of nerve injury. Alternatively, the entire cohort best illustrates the injury patterns and military-specific outcomes of subjects that have undergone humeral external fixation.

Within the subgroup of five subjects with intact preoperative radial nerve function, two of the five patients had radial nerve dysfunction documented immediately postoperatively. Thus, there was a 40% rate of radial nerve injury after external fixation in the subgroup. These two patients had pins placed outside of the safe zone. The operative reports for these two subjects were reviewed for any explanation for the lack of postoperative nerve function. For the first of these two subjects, the procedure was performed without fluoroscopy, and the pins were applied with “tissue spreading and a tissue protector.” This subject did not regain any nerve function after prolonged observation and eventually necessitated tendon transfers. For the second subject in this subset, a nerve exploration was performed when the radial nerve deficit was appreciated after external fixation, and the nerve was found to be abutting one of the pins. This subject ultimately regained full nerve function. 

A major concern when placing external fixator pins about the humerus is the safety of the radial nerve, which prompted several authors to attempt to better define the course of the radial nerve and an associated safe zone for pin insertion about the distal humerus [4-5, 7]. An additional concern in inserting pins in the distal humerus is intraarticular placement. Reichel et al. demonstrated that the joint capsule extends minimally above the transepicondylar axis but did not specifically measure this distance [7]. Several authors have demonstrated successful treatment with posterior to anterior directed distal humerus pins [8-9]. Wegmann et al. performed an anatomic study of 95 cadaveric specimens and confirmed Kamineni’s safe zone lateral to the distal humerus, as none of the specimens had a radial nerve within this zone; however, they caution that there is no true safe zone in practice as a pin that is not placed in a perfectly lateral to medial trajectory is still at risk of injuring the nerve [10]. No radial nerve was found within 2.7 cm of the center of elbow rotation (defined as the center of the epicondyles when perfectly superimposed fluoroscopically). Wegmann et al. suggest that the only safe approach for upper extremity external fixation is to make a small lateral incision, spread the soft tissues down to the level of bone, and place the pin under direct visualization while using the tissue protector [10]. Our cohort did not have any documented nerve injuries when all pins resided within the safe zones, but this safe construct was only achieved in one subject. When considering our data with the studies that have attempted to validate the safe zones, it seems that surgeons would be better served by a knowledge of where the nerve is likely to reside-a radial nerve danger zone-and always making a true approach to the humerus rather than percutaneous techniques and blind spreading.

Based on our subjects’ final dispositions (Table 2), the need for upper extremity external fixation of any kind often portends a poor outcome, as 50% of the subjects required medical retirement. Most injury patterns that require a humeral external fixator are accompanied by complex, open fractures (Table 1). The majority of elbow trauma in our combat wounded population is caused by a combination of blasts and gunshot wounds [11-12]. Rivera et al. reported on a cohort of 189 patients that sustained combat-related nerve injuries and found that all nervous injures occurred at the time of the initial insult and not during surgery [13]. Our data are in conflict with this report, as a number of subjects had documented intact radial nerve function prior to surgery and absent function after surgery. Several authors have reported on the successful use of upper extremity external fixators for a variety of techniques in the combat wounded including definitive management of fractures (including complex periarticular patterns), acute shortening of humerus fractures to obviate the need for soft tissue coverage, and immobilization of extremities after complex blast injuries to protect pedicle flaps [14-16]. Since many wounded service members receive their first surgery after sustaining injuries in an austere environment, when external fixation is used, it is commonly performed in a damage control orthopaedics setting.

Our study has several limitations. The sample size is small, which is indicative of the relatively few indications for upper extremity external fixation and the limited use in current combat care. The quality of documentation in the deployed setting is poor, and the preoperative radial nerve function was known in only half of the cohort. However, the data is useful in detailing the significant risks of upper extremity external fixation application.

There are no clearly defined absolute indications for upper extremity external fixation. Commonly accepted civilian indications include: placing an external fixator to protect a vascular repair, to maintain rigid stability in open segmental humerus fractures, to maintain reduction for an unstable elbow dislocation [14]. Of the external fixators placed in this study, 38% met these criteria. Applying these same criteria to the subjects of this study, none of the patients that had an iatrogenic radial nerve deficits would have been indicated for external fixation. In the austere environment of a deployed military setting, the only absolute indication for external fixation of the humerus is to protect a vascular repair. Patients being treated in this environment undergo provisional surgery prior to evacuation to definitive treatment centers within 24 to 48 hours, and immobilization via splinting is likely equally efficacious to external fixation without placing the radial nerve at risk during this short time period. Most humerus fractures can be treated with splint immobilization and more definitively managed at higher levels of care. We recommend documenting a neurological examination, use described safe-zones, consider constructs that span danger zones, consider optimal pin orientation, and use open approaches for pin placement. Splinting should also be considered for injuries that will be definitely managed at higher levels of care (Table 4).

**Table 4:** Recommendations

Perform and document a neurological examination prior to and after external fixation
Consider splinting injuries that will be definitively managed at a higher level of care
Have a working understanding of the danger zones for pin placement about the humerus
Use an open approach to placing pins about the distal humerus in the radial nerve danger zone
Consider placing posterior to anterior pins or using the construct to span the radial nerve danger zone

## Conclusions

Radial nerve safe zones are at a high risk of violation when an external fixator is placed in a non-conventional setting to treat injuries sustained during combat. External fixation of upper extremity injuries in combat is rarely absolutely indicated, and associated with up to a 40% incidence of radial nerve injury. Placement of an external fixator requires anatomical expertise and use of described safe-zones. Surgeons should consider pin orientation, constructs that span danger zones, and use of open approaches. Splinting should also be considered for injuries that will be definitely managed at higher levels of care.
